# The evolution of ACEs: From coping behaviors to epigenetics as explanatory frameworks for the biology of adverse childhood experiences

**DOI:** 10.1007/s40656-024-00629-3

**Published:** 2024-10-29

**Authors:** Ruth Müller, Martha Kenney

**Affiliations:** 1 Department of Science, Technology and Society (STS), School of Social Sciences and Technology, Technical University of Munich, Arcisstr. 21, 80333 Munich, Germany; 2 Department of Economics and Policy, School of Management, Technical University of Munich, Arcisstr. 21, 80333 Munich, Germany; 3 Women and Gender Studies, San Francisco State University, 1600 Holloway Ave, San Francisco, CA 94132 USA

**Keywords:** Adverse childhood experiences (ACEs), Childhood trauma, Epidemiology, Allostatic load, Epigenetics, Molecular crediblity

## Abstract

Adverse childhood experiences (ACEs) have become a topic of public and scientific attention. ACEs denote a range of negative experiences in early life, from sexual abuse to emotional neglect, that are thought to impact health over the life course. The term was coined in the CDC-Kaiser ACE Study, an epidemiological study that surveyed 17,421 adults about ACEs and correlated the responses with participants’ current health records. Shortly after the study was published in 1998, the US CDC deemed ACEs an important public health target; however, it is only recently that ACEs feature prominently in scientific and public discourses. We contend that this rise in popularity is linked to the adoption of epigenetic explanations for how ACEs affect health. Based on a literature analysis, we trace the evolution of explanatory frameworks for ACEs—from coping behaviors to allostatic load to epigenetics—and analyze how each of these explanations not only reconsiders the mechanisms by which ACEs affect health, but also who should be held responsible for addressing ACEs and how. Epigenetics provides distinctly different discursive possibilities than previous frameworks: firstly, it offers *one* distinct molecular mechanism for how ACEs work, lending “molecular credibility” to epidemiological findings; secondly, it raises the possibility of reversing the negative effects of ACEs on the biological level. This epigenetic articulation makes ACEs attractive for new actors in science and society. Particularly, it facilitates novel interdisciplinary collaborations and attracts actors in health advocacy who are interested in non-deterministic readings of ACEs that counteract stigma and support positive health interventions and healing.

## Introduction

The concept of adverse childhood experiences (ACEs) has recently become a topic of increased public and scientific attention. The term ACEs was initially coined in 1998, when lead researchers Vincent Felitti and Robert Anda began to explore the correlation between negative experiences during childhood and physical and mental health problems in later life. In a large-scale study, known as the CDC-Kaiser ACE Study, Felitti et al. (1998) asked 17,421 adults about negative childhood experiences and compared their answers with their current health records. Based on the responses, they mapped a total of ten different types of ACEs that were common in the population, including physical abuse, emotional neglect, parental drug use and having an incarcerated family member (see Table 1 for an overview of ACEs categories). They also created the “ACE score”—which has become an instrument in public health research and clinical screening: If a person reported having had one of these ten experiences, they would be assigned one point, if they reported two, two points and so forth. For example, a person who had reported experiences of physical abuse and emotional neglect would be assigned an ACE score of 2.^1^ In their 1998 article, the researchers asserted that there was an inverse dose–response relationship between a person’s ACE score and their health in later life: the higher their ACE score, the more negative physical and mental health outcomes they were likely to experience as adults. Health conditions such as depression, heart or liver disease, diabetes and cancer all were found to exhibit this dose–response relationship. An ACE score of 4 or higher was considered to put a person at high risk for future negative health outcomes. Felitti et al. (1998) suggested that the ACE score could be an important instrument for research across the medical sciences with potential relevance for clinical practice. Since the ACE score can be measured with a self-reported survey, it could easily be incorporated into medical and public health research.

**Table 1:** Categories of the ACE Score

*Abuse*
1. Psychological abuse 2. Physical abuse 3. Sexual abuse
*Neglect*
4. Psychological neglect 5. Physical neglect
*Household dysfunction*
6. Substance abuse by a close family member 7. Mental illness of a close family member 8. Witnessing of abuse of a close family member (e.g., of the mother) 9. Incarceration of a close family member 10. Breakdown of parental relationship (divorce/separation)

While Felitti and Anda proclaimed in 1998 that ACEs were a major issue of concern and an important target of intervention for public health—a fact that was also recognized by the US Centers for Disease Control at the time—it was only in recent years that ACEs received more widespread consideration in science and society. A recent bibliometric study found that out of all publications that address ACEs between 1998 and 2018, 50% were published between 2015 and 2018 (Struck et al., 2020). In the same time period, a number of popular science books and documentary films, particularly in the US context, began to discuss the relevance of ACEs for public health and individual well-being, such as the book *The Deepest Well* (2018) by Nadine Burke Harris, California’s first Surgeon General, or the documentaries *Paper Tigers* (2015) and *Resilience* (2016).

In this article, we explore the epistemic history of the ACEs framework and its recent rise in popularity.^2^ We are interested in understanding which kinds of explanations have been offered by ACEs researchers for “how ACEs work”, that is for the mechanisms through which negative experiences in childhood affect long-term health. We approach this research question from a Science & Technology Studies (STS) perspective that foregrounds the relationship between scientific knowledge and social processes. Methodologically, we perform a discourse analysis of the ACEs literature, covering the time period from 1998, when the term ACEs is coined, up to and including 2021. In our analysis, we follow Keller’s sociology of knowledge approach to discourse (Keller 2006, 2013). This approach is particularly sensitive to tracing how knowledge and social orders are discursively co-constructed.

In this article, we present the results of our analysis. Analyzing highly-cited articles and review articles that specifically discuss biological explanations for how ACEs affect health, we identify three distinctly different explanatory frameworks that emerge and gain significance at different moments in time—although they also overlap and are sometimes used simultaneously. These frameworks are: (1) individual coping behaviors (1998–2004), (2) the allostatic load framework (2005–2013) and (3) epigenetics (2014–2021). Paying attention to how knowledge and social orders are being co-produced in the scientific literature, we show that each framework not only reframes the mechanisms of ACEs but also offers different narratives about who in society should be held responsible for addressing ACEs and by what means.

With regard to the rising popularity of the ACEs framework in science and society, we argue that, while there are certainly a number of contributing factors, the emergence of epigenetics as the most recent explanatory framework for ACEs plays an important role in this uptick. As we will show, epigenetics, firstly, connects epidemiological findings with recent advances in molecular biology, inspiring new collaborations in science; secondly, it provides a simplified causative explanation for the observed correlations in the ACE Study, therefore lending “molecular credibility” (Kenney & Müller, 2017) to the ACEs framework; and thirdly, it offers the possibility of non-deterministic readings of ACEs, emphasizing the ongoing biological plasticity of human bodies and brains across the life course, which is attractive for a broader set of actors who are interested in supporting children and youth who have already experienced ACEs.

We close our article with a critical reading of what this novel epigenetic framework for ACEs makes possible and what it might obscure when creating meaningful public health interventions for childhood adversity. Our analysis thereby aims to contribute to a growing debate about postgenomic epistemologies such as epigenetics in biomedicine and their social and political implications.

## Epigenetics and epidemiology: Novel entanglements

Epigenetics is the study of changes in gene expression that do not result from changes in the DNA sequence itself (genetic mutations) but from chemical modifications on top of DNA sequences (epigenetic alterations). These chemical modifications change how the DNA can be accessed by the enzymes that read out the DNA and thereby can increase, decrease, initiate or stop the transcription of genes. Environmental epigenetics, specifically, studies how stimuli from the environment can initiate such changes to gene expression via epigenetic modifications (Feil & Fraga, 2012).

In recent years, epigenetics has become an increasingly popular explanatory framework for understanding complex biomedical phenomena that arise at the intersection of bodies and environments. For example, diet is understood to not only affect the body in terms of caloric intake, but different nutrient compositions are also believed to affect the epigenetics of the body, which can lead to metabolic conditioning or ‘programming’^3^ that can increase or decrease the body’s ability and likelihood to store calories as fat. Such conditioning is considered particularly likely to happen when organisms are exposed to certain environmental triggers—such as diet, toxicants, and social experiences—early in life, when the body is understood to be particularly ‘epigenetically plastic’.

A subfield of studies in environmental epigenetics—variously entitled ‘social’, ‘behavioral’ or ‘neuro’ epigenetics—explores the effects of social experiences, such as stress or trauma, on the epigenetic development of the body and brain, on behavior and on mental health. Behavior and mental health, in this context, are understood to be expressions of possible underlying epigenetic changes, which have resulted from social exposures. The first studies that link epigenetic changes to behavior and mental health came out in 2004, when researchers from McGill University (Weaver et al., 2004) published studies in rats that asserted that different levels of maternal grooming would affect the epigenetic profile of their pups, with less maternal grooming leading to more anxious behavior based on changes to the expression levels of a neuroreceptor in the brain. While these studies have been criticized on various grounds, e.g., their methodological soundness (c.f. Louvel, 2020; Romijn & Louvel, 2023), they are at the same time regularly referenced as foundational studies for a novel research strand within environmental epigenetics that explores the effects of social experiences in early life on later life physical and psychological health (Samaras and Müller, forthcoming). Today, there is a growing corpus of work that studies the effects of different social experiences on epigenetic profiles and life course health, utilizing both experiments in model organisms such as rats and mice as well as human cohorts (Martins et al., 2022). Social scientists have critically engaged with this emergent field of epigenetic inquiry, pointing out the potential of these studies to produce a new biosocial understanding of health and illness (e.g., Meloni, 2014; Niewöhner, 2011; Pickersgill et al., 2013), particularly regarding the multiple health effects of traumatic experience, as well as the danger of generating novel forms of reductionism and determinism, which could frame individuals who have experienced trauma as irreversibly damaged and beyond repair (Mansfield, 2012; Waggoner & Uller, 2015; Kenney & Müller, 2017).

In recent years, researchers in epidemiology who use the ACEs framework have increasingly begun to link their work to these epigenetic studies of social experience—as we will demonstrate later in this article. This is part of a larger trend in epidemiology to adopt or invoke molecular biological approaches. This move towards molecular biology can be understood as one response to what has been described as a crisis of epidemiology in the age of molecular biology. Epidemiology largely operates with correlation-based knowledge claims about health; molecular biology tends to promise causal-mechanistic knowledge (think: the “gene-for” approach). Historian of science Olga Amsterdamska (2005) details how, in the late twentieth century during the advent of molecular biology, epidemiology’s inability to offer or promise causal-mechanistic knowledge created debates within and beyond epidemiology about its validity as a scientific discipline and as a reliable source of evidence for health policy. Recent moves to relate epidemiological studies to molecular biological mechanisms could thus be understood, within this longer history, as an effort to link correlation-based findings with studies from other fields that claim to offer causal-mechanistic knowledge in order to increase their validity, authority and acceptance. The ACEs literature, as we will show, incorporated molecular biology first by drawing on the allostatic load framework and then, more fully, through adopting an epigenetic framework. As we will show, because epigenetics is a simpler explanation than allostatic load, with a farther reach across domains, it is during the third period where ACEs gains its “molecular credibility” (Kenney & Müller, 2017).

In the social sciences, the ACEs framework has received increasing attention since its inception. Some social scientists have integrated the ACE score in their work (e.g., Meinck et al., 2017). Others have expressed interest in the ACEs framework, but have suggested that the categories of the ACE score should be revised. Scholars have pointed out, for example, that the ACE score is narrowly focused on adversity in the household, and thus incapable of capturing structural inequalities, such as racism, and their health effects (e.g., Finkelhor et al., 2013; Wade et al., 2016). In STS, only a few studies have engaged with the ACEs framework so far (Filipe et al., 2021; Lappé & Jeffries Hein, 2021; White et al., 2019). Mostly, engagement in STS has been focused on those neuroscientific and epigenetic studies of early life adversity that have recently been articulated together with the concept of ACEs, without, however, necessarily discussing the ACEs framework itself (see, e.g., Pitts-Taylor, 2019 and Tolwinski, 2019). This points to an important gap in STS when it comes to the engagements with epidemiological studies, a gap that was already diagnosed by Amsterdamska in 2005.^4^ What both sets of STS studies have in common is that they critically discuss the possibly deterministic effects of biological narratives about early life adversity. In short, if ACEs are perceived as leaving biological imprints on the body, individuals who have experienced ACEs might easily be seen as biologically damaged, which can increase stigma and discrimination (Filipe et al., 2021). Furthermore, authors express concerns that if ACEs are understood as a biomedical phenomenon, solutions will likely be also located on the biomedical level, too, rather than with the wider social determinants of health (Lappé & Jeffries Hein, 2021; White et al., 2019). These are valid concerns that we share (Kenney & Müller, 2017; Müller & Kenney, 2021). Yet, to date, no STS analysis has explored (1) how the ACEs framework has emerged and evolved over time, (2) how it relates to molecular biological studies of early life adversity and (3) how the definition and biological understanding of ACEs lead to specific framings of public health problems and their solutions. We believe that such an analysis is needed to ascertain the scientific and social potential and pitfalls of the ACEs framework. This article aims to address this gap by providing an analysis of how explanatory frameworks for ACEs, i.e., explanations for how ACEs affect health, have evolved and how knowledge and social orders have been articulated together in this literature over time.

## Material and methods

In this article, we perform a discourse analysis of the ACEs literature. We have limited our analysis to articles that (1) to a significant extent discuss mechanisms by which ACEs generate negative future health outcomes *and* (2) that are either programmatic in character or have otherwise been highly cited in the ACEs literature. We compiled our sample following procedures for conducting a narrative literature review (Sukhera, 2022). While systematic reviews profess to have achieved an unbiased overview of the complete literature on a topic, a narrative review is usually smaller in scope and ambition, and acknowledges the agency of the analyst in compiling the relevant sample for the review. For this narrative literature review, we compiled a sample of 48 articles that fit our two selection criteria and analyzed them following a grounded-theory-based approach (Charmaz, 2006) that is in line with the sociology of knowledge framework for discourse analysis as set out by Keller (2006, 2013). Some authors appear repeatedly in our review, particularly Robert Anda and Vincent Felitti, key researchers in the CDC-Kaiser ACE Study. This shows that there is a core group of researchers who have been working with the ACEs concept since its inception; have been actively promoting its usefulness for science and society; and played a key part in proposing different frameworks for how ACEs affect health. Anda has also promoted the ACEs framework through other means beyond academic research and publications, e.g., through his founding of the LLC ACE Interface, an organization that disseminates information about ACEs to a variety of stakeholders in society. The development of explanatory frameworks for ACEs thus needs to be viewed as both (1) a reflection of a developing state of the art across a variety of scientific fields (e.g., epidemiology, endocrinology, neuroscience, epigenetics) and (2) an active attempt to promote this epidemiological framework by connecting it with state-of-the-art scientific research in molecular biological fields such as neuroscience and epigenetics.

## How do ACEs affect life course health? Three explanatory frameworks

In the following sections, we present our analysis of three different explanatory frameworks that have been offered for how ACEs work appearing between 1998 and 2021: (1) the idea that the behaviors people use to cope with the persistent psychological effects of ACEs, such as alcohol and drug use, create their negative health impact (1998–2004); (2) the allostatic load model, that assumed that negative experiences in early life overload the biological systems that cope with stress and create a permanent imbalance in these systems (2005–2013); (3) the epigenetic model of ACEs that builds on the allostatic load framework but introduces epigenetic modifications as the overarching molecular mediators of ACEs, as well as a stronger focus on brain plasticity and the potential biological reversibility of the effects of ACEs (2014–2021). For each model, we discuss not only their epistemic framework, but also differences and continuities in how the literature discusses who in society should be held responsible for addressing ACEs and by what means.

While we present our findings in sequential order, this is not to suggest that each explanatory framework is limited to a specific time period. In fact, prior explanatory frameworks continue to coexist with novel accounts across time periods. However, we describe specific frameworks as central for distinct time periods because important contributions to the literature invoke them as the new cutting-edge perspectives through which researchers should understand the health effects of ACEs and which should become the guiding light for future research.

### 1998–2004: Individual coping behaviors

When Felitti et al. (1998) published the results of the CDC-Kaiser ACE Study, they presented the results largely in epidemiological terms. The core of their findings is the correlation between the number of ACEs reported and negative health outcomes in adults. While they clearly reported their findings as correlations, they assumed that underlying this correlation was, in fact, a causal relationship between ACEs and ill-health in later life. Yet, the epidemiological survey methodology did not reveal any mechanisms that could support such a claim of causation. Nevertheless, Felitti et al., 1998 hypothesize about the mechaisms: they suggest that ACEs increase the likelihood of adults engaging in health-risk behaviors as a way of coping with the lasting psychological harm done by adverse childhood experiences. They write:

The linking mechanisms appear to center on behaviors such as smoking, alcohol or drug abuse, overeating, or sexual behaviors that may be consciously or unconsciously used because they have immediate pharmacological or psychological benefit as coping devices. (Felitti et al., 1998, p. 252f)

Here, so-called health risk behaviors, such as “smoking, alcohol or drug abuse, overeating, or sexual behaviors” are understood as responses to the lingering psychological trauma of ACEs. These behaviors are considered to offer short-term relief from psychological distress, however, at the price of harming health in the long run.

Proposing health-risk behaviors as a causative mechanism for the longterm health effects of ACEs is timely, as the question of how to decrease health-risk behaviors in the population in general had been receiving increasing attention in public health research in the 1990s (Metzl & Kirkland, 2010). The 1990s are, for example, the time period when bans on smoking in public buildings began in various countries, and when overweight and obesity are increasingly understood as a health risk. Markedly, Felitti’s interest in working on adverse childhood experiences was initially sparked by his work in a weight-loss study, through which he arrived at the hypothesis that childhood trauma negatively affects eating behaviours and the possiblity of weight loss (Gordon, 2023).

Articles published soon after the CDC-Kaiser ACE Study employed the framework of health risk behaviors as the mechanism for how ACEs affect health and often focused on elucidating the relationship between ACEs, a specific risk behavior and its health impacts. This quote, for example, focuses specifically on so-called sexual risk behaviors in women:

Unfortunately, we do not have data to evaluate the diverse physiological, psychological, cognitive, social and cultural mechanisms by which exposure to family dysfunction during childhood may influence subsequent sexual risk behaviors. However, it is possible that the sexual risk behaviors of individuals with histories of adverse childhood experiences represent desperate attempts to achieve intimate interpersonal connections. (Hillis et al., 2001, p. 210)

Here, Hillis and co-authors discuss “sexual risk behaviors” as negative coping behaviors that emerge in response to the harm inflicted by ACEs. This harm is considered to have left an imprint on the person through “diverse physiological, psychological, cognitive, social and cultural mechanisms,” mechanisms, which are assumed to exist, but that are not further discussed. The focus instead rests on risk behaviors that are believed to be more frequent in people who have experienced ACEs and cause their ill-health. This language, as we will discuss below, can also be highly stigmatizing (e.g. “desperate attempts”).

These risk behaviors also become the focus of the interventions that researchers propose during this early period. Authors suggest using the ACE score as a screening tool in primary healthcare to identify adults who might be more prone to engaging in health risk behavior (see, e.g., Chapman et al., 2004; Dong et al., 2003; Edwards et al., 2003). They therefore specifically target the medical community as key agents in addressing the negative health effects of ACEs. Below, Anda et al. (1999) describe how primary care physicians should think about and respond to smoking behaviors in people who have experienced ACEs:

We now come to a crucial question posed by our findings: How might persons exposed to childhood adversity benefit from the use of nicotine? Nicotine has demonstrable psychoactive benefits in the regulation of affect; therefore, persons exposed to adverse childhood experiences may benefit from using nicotine to regulate their mood. For such persons, attempts to quit may remove nicotine as their pharmacological coping device for the negative emotional, neurobiological, and social effects of adverse childhood experiences. […] Current smokers who consciously or unconsciously use nicotine as a pharmacological tool to alleviate the long-term emotional and psycho-biological wounds of adverse childhood experiences may need special assistance to help them quit. Such assistance includes recognition of the use of nicotine to modulate problems with affect, treatment of the residua of these adverse childhood experiences, and the use of nicotine replacement therapy or antidepressant medications. (p. 1657f)

Here, Anda and co-authors call for a specific type of attention to individuals with ACEs in primary care, which is for doctors to understand patients’ engagement in health risk behaviors as coping strategies and hence to tread carefully and offer specific supports when asking them to refrain from these behaviors in order to improve their health. Thus, in summation, in the initial period of publications about the life course health effects of ACEs, the focus of the literature rests (1) on individual health risk behaviors, which are understood to be coping strategies, as the main cause of the negative health effects in people with ACEs and (2) on suggestions to improve population health by identifying people with ACEs in primary care and helping them avoid or stop engaging in these behaviors through various kinds of psychotherapeutic and pharmaceutical interventions.

Towards the end of this period, however, questions begin to emerge whether engaging in health risk behaviors later on in life is the only way by which ACEs impact long-term health. In (2003), Dong et al., for example, published a study that shows that a dose–response relationship between ACEs and an increased likelihood of liver disease remains, even if the study participants have not engaged in relevant health risk behaviors, such as excessive drinking. Studies like this one gradually shift attention away from risk behaviors as mediating the health impact of ACEs in later life towards questions regarding the direct effect of ACEs on the body during childhood.

### 2005–2013: The allostatic load model

The second distinct explanatory framework began to appear around 2005. This period of publishing offers a different narrative than the first period. Firstly, we see the first introduction of an explanatory framework that uses molecular biology to understand the actions of ACEs on the body: the allostatic load and toxic stress model. Secondly, we see a shift from discussing how to provide primary healthcare for adults who have experienced ACEs to a focus on how to prevent the occurrence of ACEs in children in the first place. Thirdly, researchers increasingly begin to address public health policymakers as a relevant audience for their work, arguing that ACEs research indicates the need for preventative interventions.

In 2006, Robert Anda co-publishes a paper that argues that ACEs affect children’s biology in ways that influence their life course health (Anda et al., 2006). The title, “The enduring effects of abuse and related adverse experiences in childhood. A convergence of evidence from neurobiology and epidemiology,” indicates both a shift away from explanatory frameworks based on adult health-risk behaviors as causative and an interest in linking ACEs to findings from molecular biological fields such as neurobiology. In the following years, two new terms emerged in the ACEs literature that stand in for this new molecular biological understanding of how ACEs affect health: allostatic load and toxic stress.

Allostasis refer to the ability of a body to maintain relative stability as it encounters different environmental conditions. It thereby describes the process of short-term adaptation in order to maintain overall stability (Sterling & Eyer, 1988). The allostatic load model suggests that children’s bodies and brains can cope with adverse events up until a certain point, by biologically adapting to adversity in the moment and then returning their body and brain function to its previous state after the adverse event has ended. If certain adverse events are, however, too intense, or occur too often, a return to the previous state is no longer possible and long-term adaptive effects set in. These adaptive effects might have negative long-term health consequences. This is how Danese and McEwen explain the relevance of the allostatic load model for ACEs research in a comprehensive review article (2012):

How do adverse childhood experiences get ‘under the skin’ and influence health outcomes through the lifecourse? Research reviewed here suggests that adverse childhood experiences are associated with changes in biological systems responsible for maintaining physiological stability through environmental changes, or allostasis. Children exposed to maltreatment showed smaller volume of the prefrontal cortex, greater activation of the HPA axis, and elevation in inflammation levels compared to non-maltreated children. Adults with a history of childhood maltreatment showed smaller volume of the prefrontal cortex and hippocampus, greater activation of the HPA axis, and elevation in inflammation levels compared to non-maltreated individuals. Despite the clear limitations in making longitudinal claims from cross-sectional studies, work so far suggests that adverse childhood experiences are associated with enduring changes in the nervous, endocrine, and immune systems. These changes are already observable in childhood years and remain apparent in adult life. Adverse childhood experiences induce significant biological changes in children (biological embedding), modifying the maturation and the operating balance of allostatic systems. Their chronic activation can lead to progressive wear and tear, or allostatic load and overload, and, thus, can exert long-term effects on biological aging and health. (p. 29)

The allostatic load model was originally developed in aging research (McEwen & Wingfield, 2003), where it has been used as an explanatory model for premature aging and differing vulnerability to non-communicable diseases (NCDs) in later and late life. By introducing this framework to ACEs research, researchers begin to link old age, adulthood and childhood on the molecular level: childhood adversity overloads the allostatic system and creates unfavorable health trajectories that are detectable on the molecular level during childhood, remain into adulthood and manifest as disease at some later point in life. Events that overload the system are framed as “toxic stress”—stress that is no longer tolerable for the allostatic system, which leads to long-term changes to the body and brain. This is how a report by the American Academy of Pediatrics on “The Lifelong Effects of Early Childhood Adversity and Toxic Stress” (Shonkoff & Garner, 2012) defines toxic stress:

Whereas transient increases in […] stress hormones are protective and even essential for survival, excessively high levels or prolonged exposures can be quite harmful or frankly toxic,^39–41^ and the dysregulation of this network of physiologic mediators (eg, too much or too little cortisol; too much or too little inflammatory response) can lead to a chronic “wear and tear” effect on multiple organ systems, including the brain. (p. 235)

Here, toxic stress is defined as the amount of stress from which a biological system cannot bounce back and that, consequently, induces non-transient biological alterations in the body’s allostatic systems. By introducing the toxic stress and allostatic load models from aging research into the ACEs literature, researchers propose a new molecular link between childhood and old(er) ages.

In the literature, the emergence of this new explanatory framework is accompanied by two additional discursive shifts: (1) an increasing focus on the prevention of ACEs during childhood (rather than improving healthcare for adults with ACEs as in the first period) and, correspondingly, (2) a greater focus on addressing policymakers rather than physicians as key audiences for ACEs research. The quote below illustrates this new line of argumentation:

From both basic research and policy perspectives, confronting the origins of disparities in physical and mental health early in life may produce greater effects than attempting to modify health-related behaviors or improve access to health care in adulthood. (Shonkoff et al., 2009, p. 2252)

It is important to note that the argumentation above is inherently reliant on the new epistemic framework of allostatic load and toxic stress. By arguing that the negative health effects of ACEs are not necessarily mediated by coping behaviors in adult life, but caused by biological changes in early life, the new model shifts the window of attention away from adulthood and towards childhood—away from treatment and towards prevention. It suggests: if we intervene only in adulthood, we will have intervened too late. While a focus on prevention was also present in the literature of the late 1990s to mid-2000s and while an absence of such a discourse would indeed be surprising in any literature that deals with harm to children, we see a significant uptick in calls for preventive rather than remedial action starting from the mid-2000s.

As exemplified by the quote above, researchers tend to use a dual rhetoric when they argue for the importance of preventative action. On the one hand, they argue that basic science dictates that childhood is the best window for intervention in order to prevent ACEs from overloading the allostatic system in the first place. On the other hand, they often use an economic rhetoric to claim that this strategy would align with the interests of policymakers, who are framed as privileging the most cost-effective interventions. In this context, some researchers draw on common metrics from health economics for measuring health and life quality such as HRQoL (Health-Related Quality of Life) or YPLL (Years of Potential Life Lost; see Wahlberg & Rose, 2015 for a critical discussion of these metrics). These measurements are often used to assess what kind of public health interventions would yield the highest ‘benefit’ for the lowest ‘cost’. Researchers begin to argue that preventing ACEs is a cost-effective intervention. Corso et al. (2008), for example, write:

[P]ersons who experienced childhood maltreatment have a marginal decrease in at least 2 years of undiscounted quality-adjusted life expectancy, compared with persons who did not experience childhood maltreatment. A cost-effectiveness analysis of an intervention designed to prevent childhood maltreatment, therefore, would include 2 QALYs saved for every case of childhood maltreatment prevented. (p. 1099)

This quote illustrates the argument for ACEs prevention as a cost-effective health policy measure that is typical for the second period. Thus, at the end of the second phase of publications, ACEs are presented (1) as affecting bodies on the molecular level during childhood, thereby creating unfavorable health trajectories and (2) a target for preventive policy interventions, which can decrease healthcare costs by preventing ACEs from occurring in the first place and avoiding these unfavorable health trajectories.

### 2014–2021: Epigenetics

Epigenetics constitutes the third distinct explanatory framework for the effects of ACEs on health we identified in the literature. The epigenetics framework builds heavily on elements introduced in second phase: just like the allostatic load framework, it argues that ACEs affect life course health through molecular processes that take place during childhood. However, epigenetics introduces two new aspects: on the one hand, it simplifies the allostatic load model by offering epigenetics as the key underlying mechanism that causes biological change in response to toxic stress. While the allostatic load literature characterizes the mechanisms variously as, for example, “smaller volume of the prefrontal cortex, greater activation of the HPA axis, and elevation in inflammation levels” (Danese & McEwen, 2012), in the third phase, epigenetics is largely postulated as the central mechanism underlying all of these changes.

Epigenetics thereby offers a simplification of the arguably technically complex allostatic load framework. The narrative becomes simple: toxic stress leads to a range of epigenetic changes that modify gene expression and thereby create negative health outcomes across the life course. Take this quote from Sun et al. (2017) as an example:

Adverse childhood experiences (ACEs), including exposure to physical, sexual, and emotional abuse, physical and emotional neglect, and household stressors, such as witnessing a mother/stepmother being abused or having an incarcerated parent, are traumatic events linked to lifelong negative adult physical and mental health outcomes^1–6^ including chronic diseases,^7,8^ adult depression,^9,10^ and risk for attempted suicide.^11^ Outcomes occur through multiple routes, including epigenetic pathways whereby traumatic events can modify gene expression in the prefrontal cortex,^12^ cause inflammation,^8^ and trigger allostatic responses to stress that alter the nervous, endocrine, and immune systems^13^ […]. (p. 882f)

**Fig. 1:**
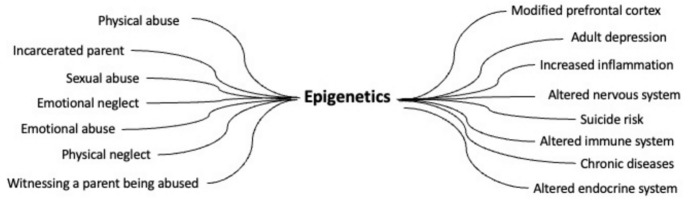
Visualization of the role of epigenetics in Sun et al. (2017). Epigenetics becomes the molecular link between various social experiences and a range of health outcomes

In this quote, a number of psycho-social experiences labeled as ACEs are said to create epigenetic changes, which lead to changes in gene expression and a range of negative health outcomes. It is important to note that in this article and many others, epigenetics is listed as *one* possible way in which ACEs can affect health outcomes as we see above: “[o]utcomes occur through multiple routes, including epigenetic pathways”. However, epigenetics is the *only pathway that is mentioned by name*.^5^ Thereby, a hypothetical multitude of pathways is, in practice, reduced to one pathway that is referred to persistently.^6^ Figure 1 below illustrates this rhetorical trope that renders epigenetics the missing link between diverse adverse childhood experiences and various health outcomes, many of which are also seen in the allostatic load model.

Notably, this new emphasis on epigenetics happens despite the fact that studies linking ACEs to epigenetic changes to negative health outcomes in humans are largely lacking today (see Jiang et al., 2019 for a review of the current state of evidence). Epigenetics is thus invoked not as a proven molecular pathway but rather as a speculative link between psycho-social experiences during childhood and later-life health outcomes.

**Fig. 2:**
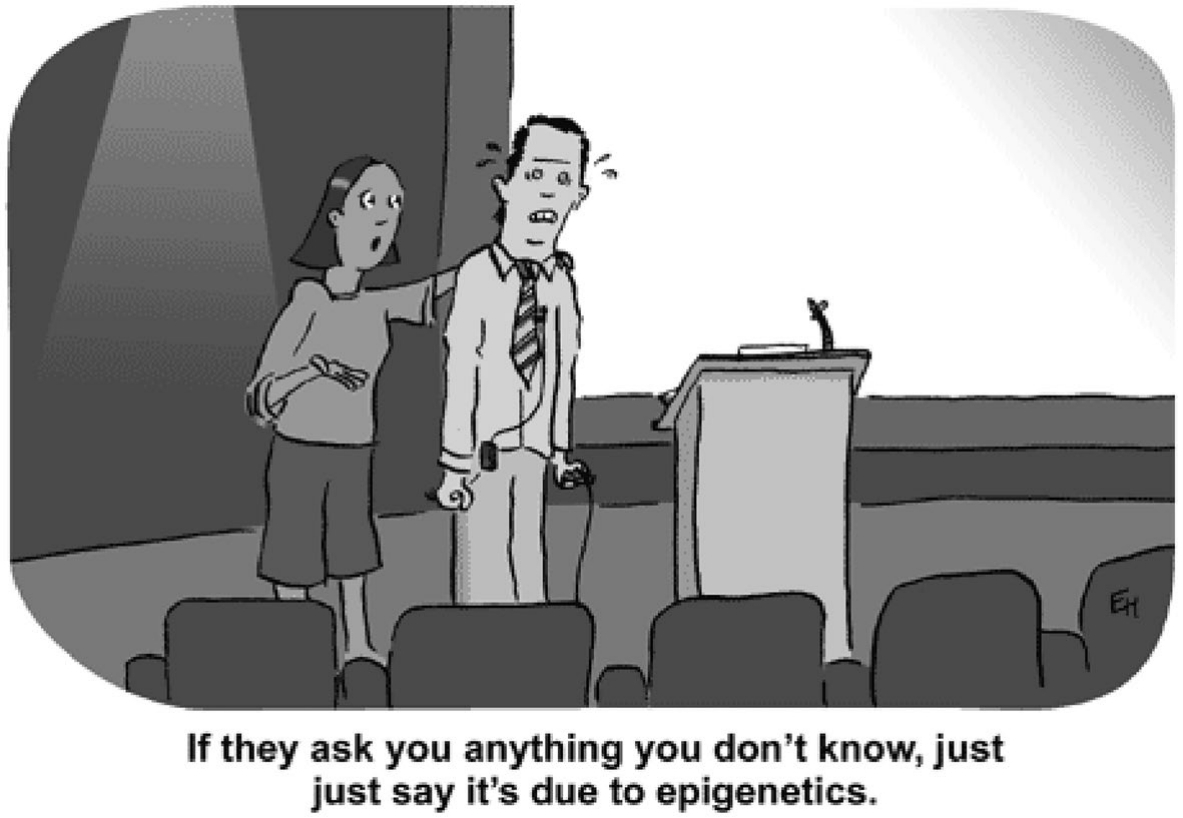
Cartoon by Ed Himelblau https://ch.promega.com/resources/pubhub/cartoons/cartoon-53/

STS researchers such as Pickersgill (2018) have drawn attention to the fact that epigenetics has often come to function as an “imagined biological” in both science and society. This is not to say that epigenetics research is not offering important scientific insights and has, in important ways, shifted the focus of research away from simplistic, gene-centric models of life, development and health. However, at the same time, it draws much of its allure from functioning as a hypothetical biological connection between material and social exposures and health outcomes—a missing link that is believed to exist even if research has not been able to elucidate it yet. Researchers in epigenetics are largely aware of this specific discursive functioning of epigenetics (Samaras & Müller, forthcoming). For example, at conferences, researchers frequently show a specific cartoon that depicts a woman, who is giving a pep talk to a colleague before a presentation, suggesting: “If they ask you anything you don’t know, just say it’s due to epigenetics” (see Fig. 2 below). However, this cartoon is usually shown as a critical footnote before a talk that posits epigenetics as the missing link between exposures and outcomes that requires further exploration. Similarly, the publications analyzed for this article invoke epigenetics as one possible pathway connecting ACEs to life course health, but through their “narrative choreography” (Müller & Kenney 2021; Kenney & Müller 2024) they, at the same time, render epigenetics the ultima ratio behind this connection.

**Fig. 3:**
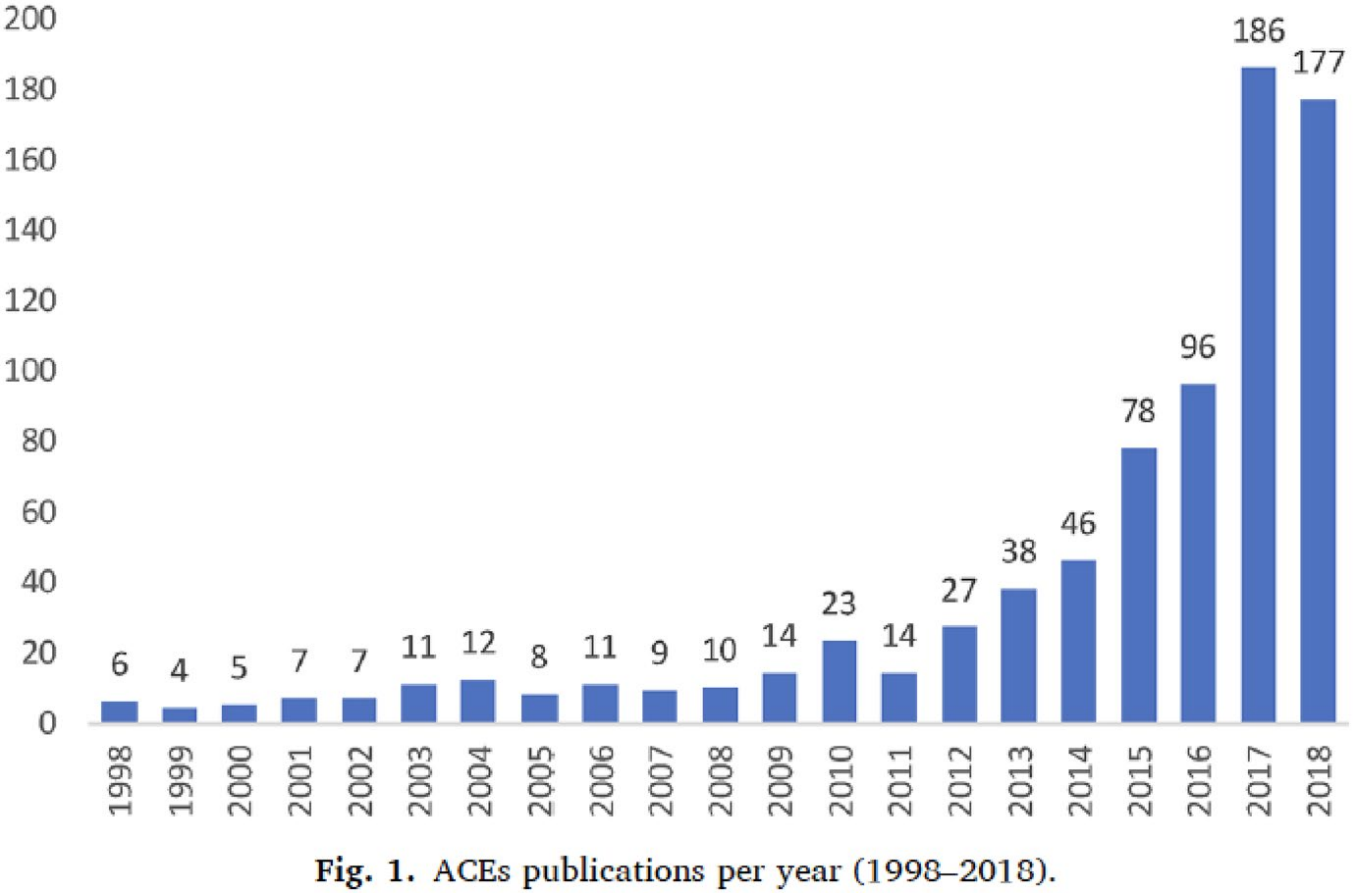
Figure 1 from Struck et al., 2020, showing the number of ACEs publications per year from 1998 to 2018

**Fig. 4:**
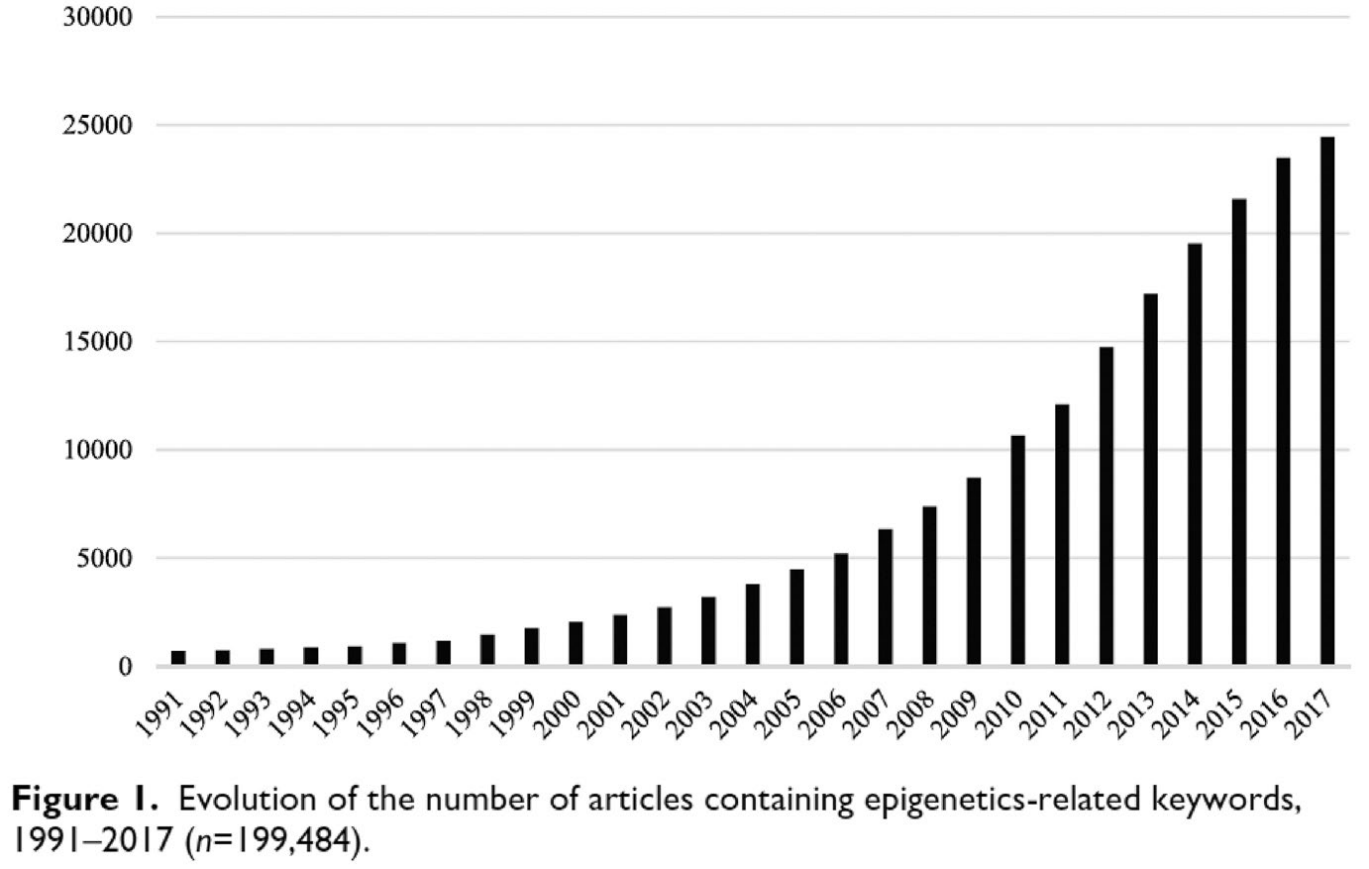
Figure 1 from Larregue et al. (2020), showing epigenetics-related articles from 1991 to 2017

The shift to epigenetics as an explanatory framework in the ACEs literature coincides with what Struck et al. (2020) found to be the period with the steepest increase in publication activity that use the ACEs framework, the period between 2015 and 2018 (see Fig. 1 from Struck et al., 2020). This increase in publications about ACEs happens at the same time as what Larregue and colleagues (2020) define, based on bibliometric analysis, as a period of “growing conceptual standardization of epigenetics research” (p. 135). They argue that between 2011 and 2017, approaches in epigenetic research have become more similar across different research objects (e.g., descriptions of epigenetic research about cancer becomes similar to those about cognitive function or plant biology). Additionally, epigenetics itself is becoming an increasingly dominant keyword, whereas earlier, epigenetics was not always present as a keyword (and instead terms such as DNA methylation or histone modification alone signified the connection to epigenetics). With Larregue et al. (2020) we interpret these shifts as an increasing consolidation of epigenetics as a research approach and explanatory framework (if not necessarily as a research field due to its heterogeneity of research objects, c.f. p. 133). Furthermore, we understand these shifts as indicative of epigenetics increasingly becoming an established term that is used as an umbrella for denoting research into a range of different forms of DNA modification without DNA mutation (c.f. Meloni & Testa, 2014). In short, the shifts indicate that epigenetics, which, historically, had been rather at the fringes of biological research, has become a “molecular biological bandwagon” (Fujimura, 1988) that an increasing number of researchers are hitching their cart to due to its simplicity and explanatory power (Figs. 3, 4).

We suggest that this growth spurt in ACEs research should be understood within the context of the rising popularity of its novel explanatory framework. While we do not suggest a causal relationship between the increase in publications about ACEs and the stabilization of epigenetics as a research approach, the literature around this time shows a bi-directional uptake in approaches: researchers working on ACEs are starting to cite epigenetics as a key explanatory framework for how ACEs affect health (e.g., Soares et al., 2021); and epigenetics researchers begin to adopt ACEs as a framework to articulate and categorize early life adversity (c.f. Jiang et al., 2019). As we have noted elsewhere (Kenney & Müller, 2017), the latter can be explained in part by epigenetics researchers looking for frameworks, such as, for example, the Developmental Origins of Health and Disease (DOHaD) framework, and more recently ACEs that have a closer relationship with healthcare and health policy, which can help them argue for the societal relevance of their work. In turn, research fields that have traditionally made claims based on correlations between exogenous factors and health outcomes, which is the case for both ACEs and DOHaD research, now often reference epigenetics as the putative mechanism behind their findings, which can “lend molecular credibility to their work” (Kenney & Müller, 2017, p. 38). Beyond mutual citations, this relationship also inspires new collaboration in research, which is evident, for example, in the author constellations of prominent articles, which tend to involve, for example, both psychiatrists and basic life scientists.^7^ For researchers in social epigenetics, the ACEs framework also offers a useful tool to capture their otherwise often ambiguous research object “early life adversity”. Working with the ACEs questionnaire, for example, allows researchers to quantify a range of experiences as an “ACE score,” enables comparability between studies, and allows them to connect their findings with a wider array of non-molecular studies that have used the ACEs questionnaire before. Furthermore, ACEs are considered an important public health target, adding social relevance to what is often otherwise considered exclusively basic research.

Beyond facilitating this confluence between the ACEs framework and molecular biological research, it is important to note that the emergence of epigenetics as an explanatory framework for the health effects of ACEs coincides with two more discursive shifts. Without necessarily making causal claims, we want to draw attention to these two shifts: firstly, with epigenetics, we find increasing references in the literature to the possibility of an ongoing biological plasticity of the brain and body and, consequently, to questions regarding the reversibility of epigenetic marks and their assumed health effects. For example, in one of his last articles before his death, entitled “In pursuit of resilience: stress, epigenetics, and brain plasticity”, renowned neuroendocrinologist Bruce S. McEwen (2016), who coined the term ‘allostatic load’ in 1993, offers a hopeful interpretation of the epigenetics of ACEs. He argues that epigenetics teaches us that “the healthy brain has a considerable capacity for resilience, based upon its ability to respond to interventions designed to open ‘windows of plasticity’ and redirect its function toward better health.” (p. 56; c.f. also McEwen, C., 2022). This narrative of “opening windows of plasticity” is interesting, since, until recently, the language of windows of plasticity was usually reserved for periods in gestation and early life that are thought to be inherently plastic. McEwen, however, argues that such windows can be (re-)opened later in life, through combinations of social, behavioral, and pharmaceutical interventions (c.f. also Boyce et al., 2021). Similarly, more technical review articles, such as Jiang et al. (2019), draw attention to epigenetic experiments in rodent model organisms that attest, for example, to the reversibility of epigenetic marks caused by social isolation through a mixture of social and pharmaceutical intervention (Mikics et al., 2018). As with most biological claims about ACEs, these windows of biological reversibility remain speculative; however, it is notable that this is becoming a more common trope in ACEs narratives in conjunction with epigenetic explanations. Whereas the ‘individual coping mechanisms’ explanation offers a way to prevent or ameliorate the effects of ACEs by avoiding or ceasing health-risk behaviors, the epigenetic explanation invites us to consider the possibility of wiping the slate clean at the molecular level. While research into reversibility is still ongoing and it remains unclear the degree to which reversibility is possible (Lloyd et al., 2023), the promise of reversibility remains an important aspect of the epigenetic framework.

We argue that it is this combination of offering a simpler molecular framework for the action of ACEs together with a narrative that suggests a possible reversibility of their effects that has significantly helped the ACEs framework to not only become increasingly popular across a range of scientific fields, but also to garner increasing attention in the wider public sphere. In recent years, a number of popular science books, for example, *The Deepest Well* by pediatrician and Surgeon General of California Nadine Burke Harris (2018), and other media, such as the documentaries *Paper Tigers* (2015) and *Resilience* (2016) as well as public-facing initiatives by universities, for example, by the Center on the Developing Child at Harvard University,^8^ have centered ACEs as a key public health challenge and target for change.^9^ What all of these popular media and initiatives have in common is that they combine a focus on prevention during childhood with a significant call for intervention and remediation in youth and adulthood. In this context, it is often stated that a positive relationship with one caring adult can not only buffer against toxic stress, but also help children heal from the negative biological effects of ACEs and build resilience (for a more in-depth analysis of such initiatives in education and juvenile corrections, see Müller & Kenney, 2021). It can be assumed that this increasing public attention and growing popularity will echo back into the research world and further increase the attractiveness of ACEs as a research topic.

### Discussion and conclusion

In this article, we have traced the development of explanatory frameworks for the effects of ACEs on life course health from the first publication in 1998 to 2021. We have distinguished three distinct frameworks that have been used to explain how ACEs work, starting with individual coping behaviors in the first period (1998–2005) to the allostatic load model (2005–2013), and finally to the current model that explains the effects of ACEs through epigenetics (2014–2021). What we aim to draw attention to with our analysis are not only the epistemic changes in the ACEs framework, but also how each of these frameworks emerged together with novel articulations of *who* should assume responsibility for ACEs and *how* they should act to ameliorate the health effects of ACEs. While we have presented our findings in a sequential order, this is not to suggest that each explanatory framework is limited to a specific time period. In fact, prior explanatory frameworks continue to coexist with novel accounts across time periods. However, the frameworks we associate with each time period are presented as the cutting-edge explanation for understanding how ACEs affect health in each time period.

In the first period, coping behaviors are framed as mediating the effects of ACEs: people with ACEs have psychological damage and thus engage in behaviors that help alleviate psychological distress, such as drinking, smoking, or drug use; but these behaviors also harm their health. Responsibility is conceptualized as individual and medical: individuals are tasked with changing their behaviors to protect their health and medical professionals with assisting them in this process. Additionally, the ACE score is recommended as a screening tool in primary healthcare to identify individuals who might be more prone to engaging in risk behaviors due to their ACEs in order to offer them psychotherapeutic or pharmaceutical interventions that decrease the likelihood of engaging in these behaviors. Although coping behaviors are understood as a result of ACEs rather than lack of willpower, this framework can be stigmatizing and place undue responsibility for change on those who have experienced early life adversity.

In the second period, the allostatic load model introduces the idea that the effects of ACEs are biologically mediated during childhood. While the bodies of children can withstand a certain amount of adversity and remain biologically unchanged, too many adverse experiences lead to toxic stress that irrevocably impedes the body’s ability to bounce back. In response, biology is reorganized in ways that create negative health outcomes over the life course. In this period, we see two key discursive shifts occur together with the adoption of this explanation: firstly, since the allostatic load model proposes that physiological damage has already occurred during childhood, prevention and not treatment moves to the center of attention. Secondly, articles begin to address health policy more frequently, calling for policy action to prevent ACEs from occurring in the first place. Based on the allostatic load model, they argue that childhood is the only time for effective intervention; although large-scale interventions to prevent ACEs might be costly, it would eventually save significant healthcare costs. Suggestions for concrete interventions mostly remain vague though. They tend to focus on the household and include interventions such as training parents in how to better care for their children as well as, though to a lesser extent, suggestions for more structural changes (such as monetary support for low socio-economic-status parents). Shonkoff et al.’s 2012 report for the American Academy of Pediatrics is representative of this kind of vague call for policy changes to improve early life conditions:

The multiple domains that affect the biology of health and development—including the foundations of healthy development, caregiver and community capacities, and public and private sector policies and programs—provide a rich array of targeted opportunities for the introduction of innovative interventions, beginning in the earliest years of life. (p. 239)

The second period of the ACEs literature thus foregrounds the effects of ACEs on biological development during childhood, effects that are understood as persistent, and on mobilizing different stakeholders, such as policymakers, to invest in preventing ACEs from occurring in the first place. However, the focus on prevention takes attention away from helping those who have already experienced early life adversity and the proposals for prevention are non-specific and, if specific, tend to focus on changing parenting behaviors and only rarely on structural change.

In the third period (2014–2021), the introduction of epigenetics as a hypothetical molecular mechanism serves to solidify the perspective that ACEs change the body during childhood on the biological level and that it is these changes that lead to negative health outcomes later on in life. Importantly, while the allostatic load model was often presented in rather technical language, epigenetics serves to simplify the biological narrative and to articulate it more easily to diverse audiences. Furthermore, it connects ACEs researchers with a growing community of epigenetics researchers who are researching the biology of early life adversity.

These shifts create new relations between ACEs researchers and actors within and outside science. Within science, ACEs and epigenetics research appear to enter into a productive relationship, with epigenetics lending molecular credibility to the ACEs framework and childhood adversity emerging as a field of application for epigenetic research approaches. Beyond science, and in some scientific circles, we believe that the popularity of the ACEs framework has risen with the addition of a final feature: the possibility of reversing the epigenetic effects of ACEs. Within the mechanistic landscape of epigenetics, biological reversibility becomes an option—an option, however, that remains speculative in the same way as epigenetics as a primary mechanism for how ACEs work is speculative, too. However, the mere *option* of biological reversibility opens up a range of novel possibilities for ACEs interventions. It brings back the possibility of treatment and healing in addition to prevention. While clinicians and policymakers remain a target audience asked to address ACEs, the possibility of biological reversibility opens doors for researchers and practitioners in fields such as education, social work, or psychotherapy, whose experiential knowledge tells them that change and healing are possible in and beyond childhood. It is this last point that leads, for example, community organizers to refer to ACEs research in its recent epigenetic iteration as a “science of hope” (c.f. Müller & Kenney, 2021). It is also the key message that books and films like *The Deepest Well* and *Paper Tigers* drive home: ACEs can be harmful, but change is possible. Many of these narratives locate the responsibility for change not on the level of the individual, as with the individual coping behaviors framework, but on the level of communities and institutions. *Paper Tigers* narrates the story of a school in the state of Washington that changed its practices to better serve children who had experienced childhood adversity. *The Deepest Well* ends with an epilogue that imagines a future in 2040, where any stigma around ACEs has been removed and “celebrities volunteer to be part of the Faces of ACEs ad campaign, and they share their story along with a call to action: *Know your score and learn how to heal*” (Burke Harris, 2018, p. 223).

As we have detailed elsewhere (Müller & Kenney, 2021), crafting these hopeful stories from the science that is currently available requires significant work. Most scientific accounts of ACEs today still operate in a rather deterministic and damage-centered mindset. Nevertheless, the most recent epigenetic instantiation of ACEs allows for different readings and offers attachment sites for novel actors, many of whom have experiential or scientific knowledge that attests to the possibility of change, learning, and healing across the life course. Whereas the individual coping behaviors framework placed the responsibility for the health effects of ACEs in the hands of individuals and their doctors; and the allostatic load model tasked policymakers with the responsibility to prevent ACEs; the epigenetic model addresses clinicians and policymakers, but also makes ACEs actionable for a wide variety of actors, including early childhood educators, psychologists, social workers, and others who work with families, children, and communities. As anti-deterministic readings of ACEs have become supported, at least in part, by scientific research, the popularity of the ACEs framework has risen significantly.

While initiatives based on an epigenetic understanding of ACEs are new, this novel, speculative epistemology opens opportunities for new partnerships between those who study ACEs and those who work with children and adults who have experienced ACEs. It is possible that the ACEs framework will undergo yet another epistemic change and the possibility of determinisms always looms large. However, we suggest that it would be generative to take advantage of this moment to collectively consider what kinds of knowledge, practices, and interventions are being enabled by foregrounding plasticity, biological reversibility and the ongoing possibility of change across the life course. Here, research could take a cue from practitioners who work with children and youth with ACEs and who hold important knowledge about how change and healing can be facilitated in practice. This knowledge could be vital to the design of future research projects and epistemic agendas that explore the biology of ACEs from a non-deterministic and healing-centered point of view.

## Footnotes

1 Importantly, multiple instances of the same ACE only count as one point in the ACE score. Robert Anda has noted this limitation himself, stating that “the questions from the ACE study cannot fully assess the frequency, intensity, or chronicity of exposure to an ACE…or differences in the timing of exposure.” (Anda et al., 2020, p. 293).

2 We are aware that ACEs is one framework among many that tries to capture the effects of adversity on the body and mind and that it is part of an extensive history of thinking about trauma and adversity in psychiatry, medicine, psychology, philosophy and the social sciences. Analyses of aspects of this extensive history have been published in this journal (most recently, e.g., Lloyd et al., 2022) and elsewhere (e.g., Leys, 2010). While we are aware of this complex history, in this article, we focus exclusively on the relatively short history of the ACEs framework itself, which deserves detailed attention due to its growing prominence across scientific fields and societal arenas.

3 See Hanson and Gluckmann (2014) for a critique of the term ‘programming’ from within biomedical research; see Richardson (2015) and Kenney & Müller (2017) for critiques from the social sciences.

4 There are many possible explanations for this gap: one could be that STS research is often tied to funding lines for emerging technologies and research fields. In biomedicine, these have generally been research fields within molecular biology, such as genetics, neuroscience, nanotechnology or epigenetics. Case in point are the ELSI/ELSA (Ethical, Legal and Social Issues/Aspects) programs that emerged with the Human Genome Project, and were later expanded primarily to other molecular and high-tech driven fields of research, such as neuroscience or nanotechnology (Hilgartner et al., 2017).

5 A second mechanism that is sometimes mentioned by name is telomere length (e.g., Bürgin et al. 2019). However, epigenetic processes and processes determining telomere length seem to be interdependent, though the nature of their interdependency is not yet clearly elucidated (Criqui et al. 2020).

6 Readers familiar with epigenetics will note that epigenetics itself consists of a range of molecular mechanisms, such as methylations, acetylation or RNA modification. This diversity of mechanisms—and disputes about which should be labelled epigenetic and which not—is not widely discussed in the ACEs literature. Rather, epigenetics is presented as one distinct and clearly defined phenomenon.

7 For example, this is how the list of affiliations of the Jian et al. (2019) review article “Epigenetic Modifications in Stress Response Genes Associated With Childhood Trauma” reads: Shui Jiang^1^, Lynne Postovit ^2^, Annamaria Cattaneo^3^, Elisabeth B. Binder^4,5^ and Katherine J. Aitchison^1,6*^ ^1^ Department of Medical Genetics, University of Alberta, Edmonton, AB, Canada, ^2^ Department of Oncology, University of Alberta, Edmonton, AB, Canada, ^3^ Biological Psychiatric Unit, IRCCS Istituto Centro San Giovanni di Dio Fatebenefratelli, Brescia, Italy, ^4^ Department of Translational Research in Psychiatry, Max Planck Institute of Psychiatry, Munich, Germany, ^5^ Department of Psychiatry and Behavioral Sciences, Emory University School of Medicine, Atlanta, GA, United States, ^6^ Department of Psychiatry, University of Alberta, Edmonton, AB, Canada.

8 https://developingchild.harvard.edu/science/

9 While this wider public attention to ACEs in its epigenetic instantiation is particularly pronounced in the US, Canada and the UK, it can be noted that it is gradually spilling over into other national contexts, particularly through traditional media (books, films) and social media networks. Robust studies about transnational attention to ACEs though are currently missing in the literature.
